# Dredging Activities Carried Out in a Brazilian Estuary Affect Mercury Levels in Swimming Crabs

**DOI:** 10.3390/ijerph17124396

**Published:** 2020-06-18

**Authors:** Paloma de Almeida Rodrigues, Rafaela Gomes Ferrari, Rachel Ann Hauser-Davis, Luciano Neves dos Santos, Carlos Adam Conte-Junior

**Affiliations:** 1 Molecular and Analytical Laboratory Center, Department of Food Technology, Faculty of Veterinary, Universidade Federal Fluminense, Niterói 24230-340, Brazil; paloma_almeida@id.uff.br (P.d.A.R.); conte@iq.ufrj.br (C.A.C.-J.); 2 Chemistry Institute, Food Science Program, Universidade Federal do Rio de Janeiro, Rio de Janeiro 21941-909, Brazil; 3 Laboratório de Avaliação e Promoção da Saúde Ambiental, Instituto Oswaldo Cruz, Fundação Oswaldo Cruz, Rio de Janeiro 21040-360, Brazil; rachel.hauser.davis@gmail.com; 4 Laboratory of Theoretical and Applied Ichthyology, Department of Ecology and Marine Resources, Universidade Federal do Estado do Rio de Janeiro, Rio de Janeiro 22290-240, Brazil; luciano.lep@gmail.com; 5 National Institute of Health Quality Control, Fundação Oswaldo Cruz, Rio de Janeiro 21040-900, Brazil

**Keywords:** Guanabara Bay, mercury, bioindicator, abiotic variables, dredging

## Abstract

(1) Although suffers from intense pollution inputs, Guanabara Bay, the most socioeconomically and environmentally important estuary in Rio de Janeiro, Brazil, is still home to a diverse fauna, including several fish and crab species consumed by humans. The bay presents high sedimentation rates and sediment contamination, further aggravated by dredging processes carried out in recent years. In this context, this study aimed to verify the effect of the dredging process on total mercury (THg) concentrations at Guanabara Bay through swimming crab assessments sampled before (2016), during (2017), and after (2018) the dredging process, and mainly, if the detected concentrations can be harmful to consumer health; (2) Methods: Swimming crab samplings were carried out at the same time and sampling points in 2016, 2017 and 2018 and the total Hg was determined using a Direct Mercury Analyzer (DMA-80, Milestone, Bergamo, Italy); (3) Results: Increased Hg concentrations were observed during the dredging process, decreasing to lower values, close to the initial concentrations, at the end of the process. Some of the investigated abiotic factors favor Hg dynamics in the aquatic environment, while others were positively altered at some of the assessed sampling areas at the end of the dredging process; (4) Conclusions: Although crab Hg levels were below maximum permissible limits for human consumption, it is important to note that these animals are significantly consumed around Guanabara Bay, which may lead to public health issues in the long term.

## 1. Introduction

Guanabara Bay (GB), a eutrophic estuary located in the state of Rio de Janeiro, southeastern Brazil, displays significant economic, social and ecological importance, sustaining a significant diversity of aquatic organisms, including fish, crustaceans, mollusks, cetaceans, polychaetes and algae. It is, however, highly impacted by pollution originating from domestic and industrial sewage discharges, which contains, among other, high levels of polycyclic aromatic hydrocarbons, pharmaceutical drugs and metals, including mercury (Hg) [1,2]. Despite the existence of other potentially toxic metals at GB, mercury (Hg) is a contaminant of worldwide interest, due to its global importance, being one of the only metals directly implicated in an excessive number of human deaths.

The main sources of mercury in sediment, water and living organisms are related. There are natural atmospheric sources, related to the elementary mercury form that has a high capacity for volatilization, such as emanations from atmospheric volcanoes, continental degassing and evasion of Hg from the oceans. This form of the volatilized metal precipitates with rain and reaches the aquatic environment. In addition, another natural source is weathering that favors the release of mercury that is carried into the water. Hg inputs for GB are mainly due to anthropogenic activities, such as dumping of alkali chloride plants, metallurgical residues, paint industry, burning fossil fuels and domestic sewage [1,2,3,4,5]. The mercury, before it reaches the water, is usually in inorganic or elemental form. The elemental form upon reaching water undergoes oxidative processes and combines with elements such as chlorine, sulfur or oxygen, becoming inorganic mercury. The metal can then be adsorbed by particulate matter in suspension and brought to the sediment by flocculation or sedimentation. Mercury can be resuspended from the sediment, making it bioavailable for absorption by living organisms. It also becomes available to be methylated by sulfate-reducing anaerobic bacteria, forming methylmercury (MeHg), which is dispersed in water, having high bioavailability. The process of resuspension of sediments occurs mainly in response to the movement of water, when, for example, a current or waves exert a shear force on the sediment. However, dredging is also a process that promotes the resuspension of sediments, which occurs during its mechanical removal [6,7,8,9]. As GB presents high sedimentation rates (0.6 cm year^−1^ to 4.5 cm year^−1^) [10], mostly as a result of the significant deforestation of the drainage basin and river channeling, the local Rio de Janeiro authorities carried out a dredging process from June 2016 to November 2017, near the Rio de Janeiro seaport. The dredged sediment was removed from this area and released into the oceanic region adjacent to the bay. This aimed to minimize problems associated with contaminant accumulation, increasing vessel access channel depth, improving water circulation and coastal water renewal, promoting macrodrainage for the disposal of surface waters, consequently improve water oxygenation and improving operating hydraulic work conditions, and controlling critical hydrological events [11]. Thus, dredging may in fact worsen Hg contamination by making this element bioavailable in its most toxic form. Both estuarine and coastal environments, due to anthropogenic action, present accelerated methylation rates, due to several variables, including anoxia, high levels of organic material and sulfate and favorable dynamics between sediment and water, [6,12]. In addition, abiotic factors such as pH, temperature, dissolved oxygen, among others, directly affect Hg bioavailability in the aquatic environment and consequently its concentrations in animal tissues, requiring constant monitoring [5,13].

Hg displays the ability to biomagnify throughout the food chain from the lowest trophic level organisms to humans, who are intoxicated mainly by the consumption of these contaminated seafood [6,14,15,16,17]. The animals have as their source of contamination the metal dispersed in the water, but mainly the one that is in the sediment and that is sometimes resuspended, being absorbed through gills, and especially, through the dietary route, accumulating in different tissues, especially hepatopancreas, the organ responsible for detoxifying metals [7,15,16]. It is a potent neurotoxin, with the potential to affect different body systems, alter reproduction rates, increase mortality rates and, finally, cause ecological imbalances [17,18].

The main fishing activities at GB is artisanal fishing [4]. In addition to fish, swimming crabs, constitute a significant portion of artisanally captured, marketed and consumed protein sources in the area, totaling about 126 t between 2005 and 2007 [19]. Thus, Hg contamination in dietary items from GB consumed by humans should be regularly monitored, in order to assess public health risks to the local human population. Swimming crabs are so named because they display a modified last pair of legs that, instead of being pointed, are wide and flat, giving them swimming flexibility. They are benthic organisms that live in close contact with potentially contaminated sediments and are, therefore, considered important contamination bioindicators [4,6,14]. Studies around the world that evaluate the concentration of mercury in swimming crabs that inhabit estuaries indicate average values between 0.035 mg kg^−1^ and 0.496 mg kg^−1^ [5,6,20,21,22,23,24,25,26].

Based on the assumption that dredging objective to remove sediments that are known to be contaminated by Hg, our aims in this work were: (1) to prove the hypothesis that contamination by this metal would be reduced with dredging, by checking the total concentrations of Hg in swimming crabs, determined before, during and after dredging; (2) to evaluate the abiotic data of the water (pH, dissolved oxygen, temperature, salinity, depth, transparency) to correlate them with the findings of total mercury in the investigated swimming crabs; and finally, (3) to verify if the Hg contamination in swimming crabs found in the study can lead to potential risks to the consumer’s health.

## 2. Materials and Methods

### 2.1. Study Area

**Figure 1:**
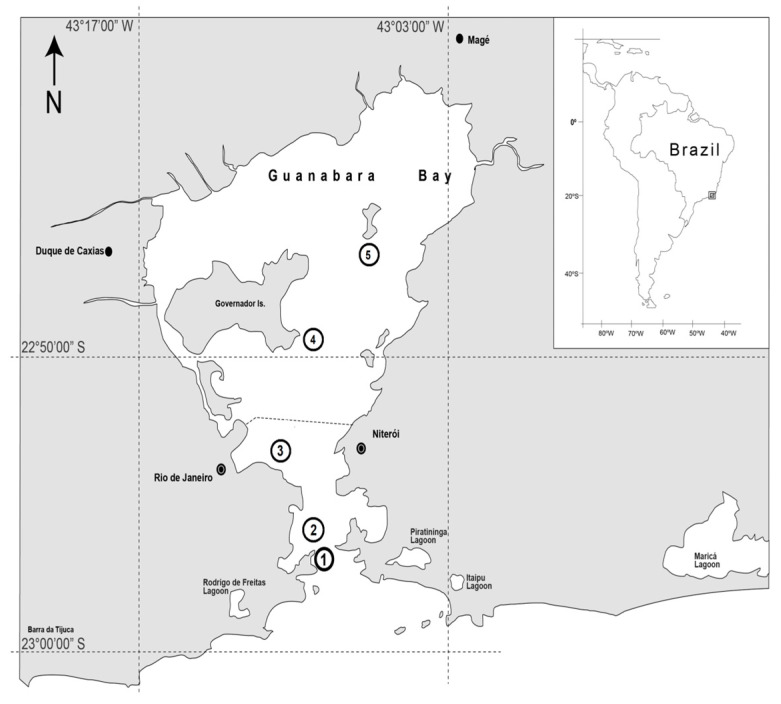
Guanabara Bay. Swimming crab sampling points: P1, Urca Beach; P2, Flamengo Beach; P3, Seaport of Rio de Janeiro; P4, Engenho (São Gonçalo); and P5, Paquetá Island.

Guanabara Bay (22°24′–22°57′ S, 42°33′–43°19′ W) comprises a total area of 4081 km^2^, with an estimated water volume of 1.87 billion m^3^. Five sampling points were assessed in the present study, in order to contemplate the entire estuarine gradient: Urca Beach (P1), Flamengo Beach (P2), both external bay points; the Rio de Janeiro seaport (P3), in the middle of the bay, and Engenho (P4) and Paquetá Island (P5), both internal bay points (Figure 1).These points were chosen considering vessel flow (Porto), effluent disposal (all points, specially Engenho and Paquetá), leisure areas and tourist points (Urca beach, Flamengo and Paquetá) and local fishing activities (all points, specially Urca and Porto).

Although dredging was only carried out at the port, the adjacent external and internal areas were also assessed since, as an estuary, the water flow from rivers and the open sea promotes constant water changes in the region. Thus, by evaluating the adjacent areas, potential dredging effects on contamination in nearby areas were also investigated.

Paquetá Island is situated in the inner zone of the bay, at the northward limit of the main water circulation channel, still under the influence of marine waters. However, water circulation is not as high as in the southeastern portions of the bay due to decreased tidal current speeds. Therefore, this region displays low water renewal rates and is the target of several pollution sources [10,13,27]. Engenho is located in the municipality of São Gonçalo, considered as an interior bay region. Due to the significant population and industrial growth, this area is impacted by both domestic and industrial sewage discharges, aggravated by the low water renewal rates, due to its internal location [10]. Flamengo and Urca Beach are considered external seaport points since they are located near the oceanic zone, mainly Urca. Urca Beach is located very near the mouth of the bay, in the lower estuary region, suffering the influence of both oceanic marine waters and organic matter from inside the bay [13,27,28]. The Rio de Janeiro seaport is the major local domestic and international cargo tourist and people transportation route by the sea. Thus, it is intensely contaminated and displays high sedimentation rates, which leads to decreased local depths and vessel berthing problems [11,13].

### 2.2. Swimming Crab and Water Samplings

Swimming crab samples were collected in the third week of March in the years 2016, 2017 and 2018 at the selected sampling points. No swimming crabs were found at the Rio de Janeiro seaport in 2017, which may have happened due to the dredging process, with the removal of the sediment together with the animals. A total of 10 bottom trawls were carried out at each sampling site using a single-port net (25 m net mouth and 6 m high, 25 mm mesh between adjacent nodes). Two trawls, each with a standardized total duration of 20 min, were carried out at each of the collection sites. The animals collected were kept on ice until arriving at the lab, where they were then frozen at −20 °C until the moment of analysis biometrics (species identification, length and sexing) and quantification of total mercury.

After collecting the animals, water samples from each point were collected at 1 m from the bottom with the aid of a Van Dorn bottle, and abiotic parameters (pH, salinity (g L^−1^), temperature (°C) and oxygen levels (mg L^−1^) were immediately determined in the field with the collected samples using a multiparameter water quality meter (Multiparameter Probe HI9828, Hanna Instruments, Póvoa de Varzim, Portugal). After checking the parameters on the Van Dorn bottle, the water was discarded. Water transparency was assessed with the aid of a Secchi disk, while depth was obtained by using a GPS.

### 2.3. Biometric Swimming Crab Processing

Swimming crabs were classified according to genus and species, based on phenotypic characteristics described in the literature [29,30]. The collected species were identified as *Callinectes spidus*, *Achelous spinimanus* and *Achelous spinicarpus*. Animals were sexed when possible. In 2016, a total of 44 animals were collected, 25 in 2017 and 40 in 2018, totaling 109 animals. Regarding sex, a total of 25 females and 19 males were sampled in 2016, 16 females and 9 males in 2017, and 11 females and 29 males in 2018. Total carapace lengths (mm) with caliper were determined, and only medium to large animals (approximately 70–80 mm) were selected, corresponding to sizes destined for human consumption. The animals had their carapace removed manually to collect the muscle tissue located inside the carapace, for the total Hg determinations.

### 2.4. Mercury Analysis

#### 2.4.1. Sample Analysis

Analyses were performed in triplicate. Before each sample weighing, the sample crucibles were washed with soap and rinsed with distilled water, dried, and heated at 650 °C for 3 min in a DMA-80 Direct Mercury Analyzer (Milestone, Bergamo, Italy), to avoid contamination or interferences. Before each sample analysis, blank values were verified to be always less than 0.001 Hg (ng) [31].

Total Hg in the swimming crabs was determined following the manufacturer’s recommendations. Briefly, about 0.2700 g ± 0.0030 g of each pooled muscle sample were inserted in a quartz tube and dried under an oxygen stream at 160 °C for 1 min, 650 °C for 2 min, and 650 °C for another 1 min, at 4 atm. After drying, the Hg vapor was then desorbed using a gold amalgamation trap and Hg was pre-concentrated from the flow of decomposition products. The detection system contains an Hg-specific lamp, a low-pressure Hg vapor lamp, which emits light at a wavelength of 253.7 nm and a silicon UV diode detector that was used to quantify Hg. This system comprises a dual cell spectrophotometer that affords an extremely wide detection range. Peak height was used for signal evaluation and the results were expressed as mg kg^−1^ [31].

#### 2.4.2. Calibration Curve

Standard Hg solutions were used to prepare a calibration curve from a 1000 mg L^−1^ Hg stock solution (Sigma-Aldrich, São Paulo, Brazil), ranging from 0.5 to 1000 ng g^−1^, taking into account sample weight and peak height. These solutions were used to build the ten-point calibration curve (0–15 ng g^−1^; *y* = 22.085 × *x* − 0.3217; *r*^2^ = 0.9992). In addition, confirmation tests for calibration curve were carried out during each run.

#### 2.4.3. Optimization of Analytical Conditions 

Respecting the manufacturer recommendation conditions for Hg determination, analytical parameter optimization was initially carried out. A set of experiments was performed to obtain the analysis program for Hg determination in swimming crab muscle tissue. Optimization was performed by applying three parameters: limit of quantification (LOQ), limit of detection (LOD) and accuracy. The LOQ and LOD were determined as recommended by 2011/836/EU Regulation, and the methodology validation was based on Torres et al. [32]. Method accuracy was verified by the analysis of a standard reference material IAEA-476. All determinations were performed in duplicate.

#### 2.4.4. Human Consumption Risks

Human consumption risks were calculated using the provisional tolerable weekly intake (PTWI) values stipulated by WHO and NRC (0.004 mg and 0.0007 mg kg^−1^, respectively), the average weight of the population of the state of Rio de Janeiro (70 kg), an average weekly amount of consumed crab (0.356 kg) based on literature data concerning weekly seafood consumption in the study area [19] and the Hg concentrations detected herein. The calculation was performed as follows: the first objective was to determine the weekly intake limit for a person with an average weight of 70 kg. For this, we multiplied the value of the limits established by JECFA and the NRC, which had the unit of measurement previously changed to mg kg^−1^, by the average weight of the population. Then, we multiplied each of the average values of mercury in the internal areas of Guanabara Bay, in the port and in the external areas by the average quantity of crabs consumed weekly (0.356 kg). The results of this last calculation were compared with the limit values established in the first calculation.

### 2.5. Statistical Analyses

A principal component analysis (PCA) was applied to the abiotic data matrix of the five sampling points, in order to evaluate temporal and spatial changes of the variables. Data were log-transformed and comparisons between observed versus randomized eigenvalues (i.e., AVG-RND criterion) were used to test the PCA significance and to retrieve the significant axes for analysis. The PCA analyses were performed using the PC-ORD 6.0 statistics package (Wild blueberry media LLC, Corvallis, OR, USA).

Two constrained redundancy analyses (RDA) were carried out with the aim of verifying potential relationships between abiotic factors, Hg concentration and animal lengths, first grouping the sampling points as external and internal to the bay, and second, by considering the periods before, during and after dredging. RDA analyses were performed using the CANOCO 4.5 package (Petr & Majka Smilauer, Ithaca NY, USA).

A covariance analysis (ANCOVA), using the STATISTICA 11.0 program ((StatSoft Inc., Tulsa, OK, USA) was also carried out, using log10 transformed animal length as the co-variable. The first aim was to verify possible differences in Hg concentrations at the beginning and after sediment dredging at P3 (Rio de Janeiro seaport). Subsequently, the ANCOVA was applied to verify potential differences between the beginning, during and after the dredging in both the internal (Engenho and Paquetá) and external sampling points (Urca and Flamengo). Statistical significance was accepted at *p* < 0.05.

## 3. Results

### 3.1. Abiotic Variables

The means and standard deviations of each of the assessed abiotic variables (depth, transparency, pH, oxygen, salinity, and temperature) are displayed in Table 1.

**Figure 2:**
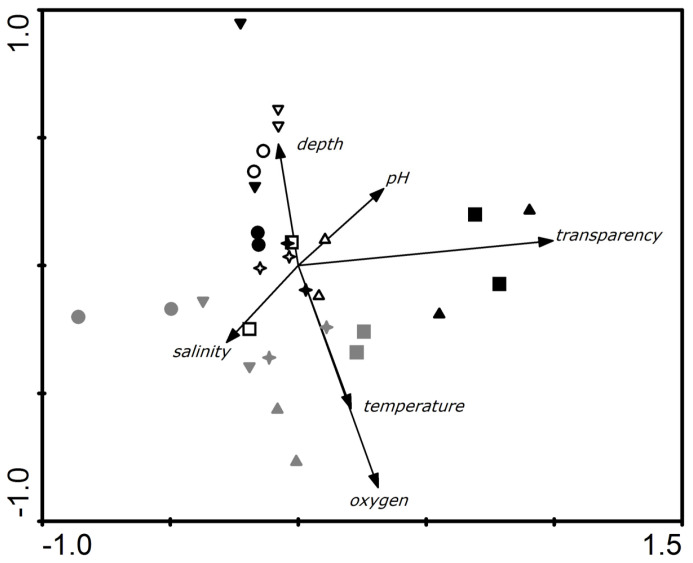
PCA carried out with the abiotic data, where axis 1 is horizontal and axis 2, vertical. Label: white symbols: pre-dredging (2016); gray symbols: during dredging (2017); black symbols: post-dredging (2018). ▲, Urca Beach; ■, Flamengo Beach; ●, Engenho; +, Seaport; ▼, Paquetá.

The first two PCA axes were retrieved for analysis (observed eigenvalues = 1.88 and 1.67 > randomized eigenvalues = 2.41 and 1.71 for axes 1 and 2, respectively), explaining 83.7% of the total data variance (Figure 2). Axis 1 was responsible for explaining 64.1% of variation, and axis 2 for 19.6% However, a significant difference (*p* = 0.0014) was obtained only in axis 1, where depth was negatively correlated to temperature and oxygen levels. Thus, the higher the oxygen concentration and temperature, the lower the depth. Salinity was also negatively correlated to transparency and pH. Thus, the higher the pH and transparency, the lower the salinity. However, temperature and oxygen were positively correlated. Some variables could be distinguished in each of the three assessed dredging periods. Depth can be highlighted at the beginning of the dredging, specifically at Engenho and Paquetá. The other points, according to the PCA, did not vary as much as the abiotic factors, since they remained grouped in the central region. During the dredging, the points were distributed around salinity and temperature. In the post-dredging period, the transparency of the water in Urca and Flamengo was evident, since the other points are located around the center. In the port of Rio de Janeiro, no significant variations were observed before, during and after dredging.

### 3.2. Swimming Crab THg Concentrations and Correlations to Abiotic Factors

Three species of swimming crabs commonly marketed in the Rio de Janeiro metropolitan area were identified herein, all displaying similar habitats and feeding habits. Our previous study, which assessed the influence of the season on Hg concentrations in swimming crabs in Guanabara Bay, found that the difference between species, as well as sex, did not influence mercury concentrations [5]. Therefore, no assessments concerning species or sex were carried out.

**Figure 3:**
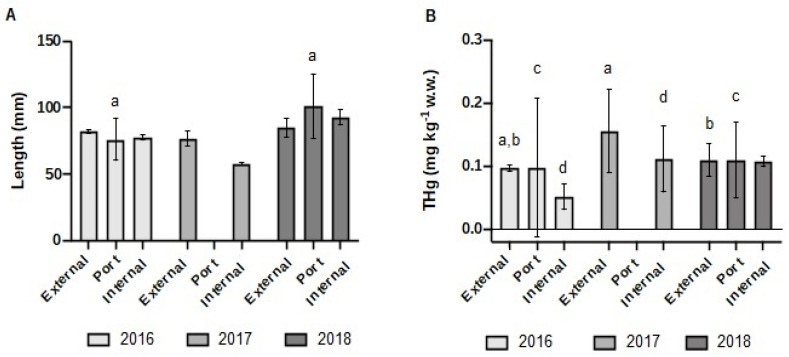
(**A**) Length and (**B**) THg concentrations in swimming crabs before (2016), during (2017) and after (2018) the dredging activities at the Rio de Janeiro seaport, external (Urca and Flamengo) and internal (Engenho and Paquetá) swimming crab locations. Same letters in columns indicate statistically significant differences between years.

There were no statistically significant differences in the length of the animals during the periods evaluated for the two external sampling points and two internal sampling points (*p* = 0.10) (Figure 3). Thus, the data were grouped by external (Urca and Flamengo) and internal (Paquetá and Engenho) locations. In the port of Rio de Janeiro, only the values of length and THg were obtained before and after dredging, as no animals were found in 2017 in this sampling site, probably due to the impact of dredging.

We did not find a significant difference in the mercury concentrations of the animals, between the external and internal points (*p* = 0.19), however we identified a significant difference (*p* = 0.05) when we evaluated the temporal variation of THg—that is, between the periods before, during and after dredging (Table 2). The previous concentrations were lower at both points, when compared to the values during the dredging period, when we identified the increase in THg. In the post-dredging period, we identified the reduction of the metal; when compared to the period during the dredging (2017), however, the concentration values were still not lower than the initial values (2016).

**Figure 4:**
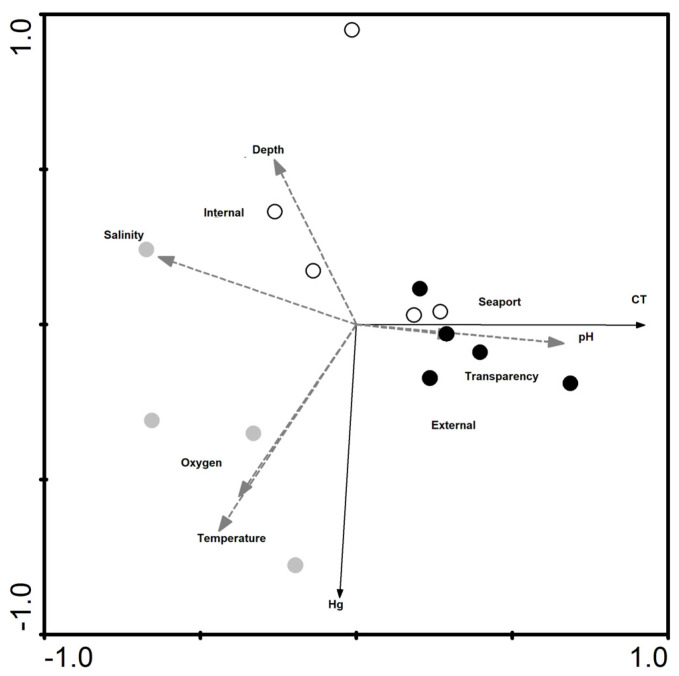
RDA performed using abiotic data (Depth, salinity, oxygen, transparency, pH, temperature), Hg concentrations and length (CT) in swimming crabs, taking into account mainly period (before, during and after the dredging) and secondarily the sample region, dividing in Seaport, external and internal areas of the Guanabara bay. The circles represent the collection points. White circles represent the collection points prior to dredging, gray during and black, after.

The RDA for mainly the periods (Figure 4) indicated that axis 1 is the most significant, explaining approximately 95.8% of the data. The *p* and *F* values were, respectively, 0.0781 and 22.200 for this axis in the Monte Carlo test. Depth was negatively correlated with oxygen and temperature, while salinity was negatively correlated with pH and transparency. Hg concentrations were negatively related to depth, where the deepest areas had less Hg, especially during the dredging period (2017), and were positively correlated with oxygen and temperature, where higher oxygen and temperature values favor the highest Hg content. It was possible to make some space-time correlations: first, we identified that the greatest depths and salinities were observed before the dredging (2016), mainly in the internal sampling points (Paquetá and Engenho). Higher pH and transparency were observed in the post-dredging period (2018), as well as an increase in the length of the animals, mainly at external sampling points (Urca and Flamengo).

Hg consumption levels through swimming crab intake in the internal, port and external GB sampling locations are displayed in Table 3.

**Figure 5:**
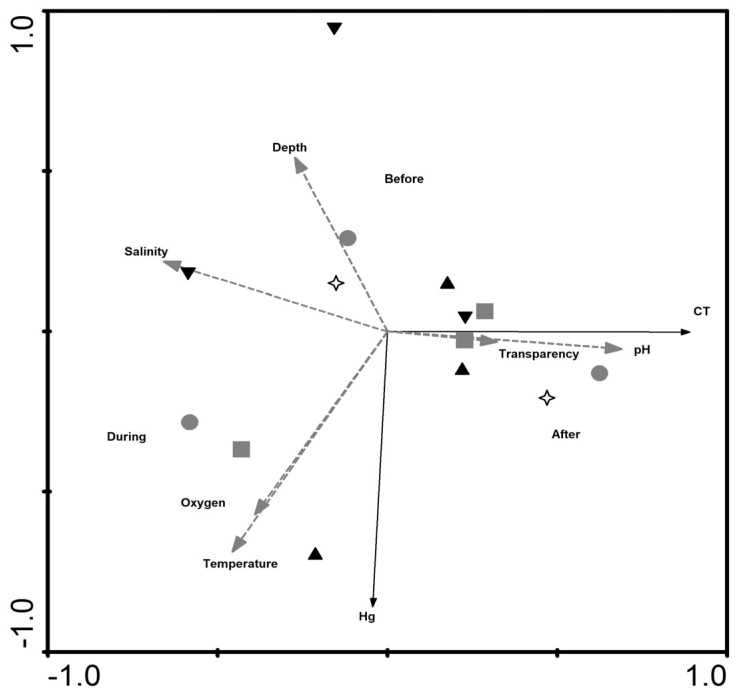
RDA performed using abiotic data (Depth, salinity, oxygen, transparency, pH, temperature), Hg concentrations and lengths (CT) in swimming crab taking into account mainly sample points and secondarily the period of dredging, dividing in before, during and after. Black symbols represent the more internal (Paquetá) and external areas (Urca), while gray indicates the other sampling points, ▲, Urca Beach; ■, Flamengo Beach; ●, Engenho; +, seaport; ▼, Paquetá.

The RDA referring to the collection point mainly (Figure 5) indicated that axis 1 is the most significant, explaining about 95.7% of the data. The values of *p* and *F* were 0.1734 and 15.606, respectively for this axis in the Monte Carlo test. The same behavior of the abiotic variable is seen in the second RDA. Greater depths were observed mainly in Paquetá and Engenho, before dredging (2016). During the dredging, the concentrations of oxygen, temperature and Hg in Engenho, Flamengo and Urca were high, while the pH and the length of the animals increased after the dredging in the port of Rio de Janeiro and Engenho. Transparency was higher after dredging in Flamengo, Urca and Paquetá.

## 4. Discussion

### 4.1. Abiotic Variables

The abiotic factors assessed did not vary significantly over the study period. When assessed by zone, the innermost regions were similar to each other and differed from the outer regions, with the port of Rio de Janeiro as the midpoint.

The port region of Rio de Janeiro was expected to have greater depth after dredging, as this was one of the main objectives of dredging. This, however, was not observed. On the contrary, the depth was not changed between the dredging period and after the process. We believe that the low depth of the studied regions, mainly the port, can be attributed to high rates of sedimentation and accumulation [10], which can overcome the sediment removal effect. According to Fistarol et al. [10], the maximum depth in most of the bay is less than 10 m, while the central channel, which undergoes greater oceanic movement of water, reaches depths of up to 58 m. For this reason, Paquetá has greater depth, since the island is located in the inner part of the bay, at the northern limit of the central channel, which is the main channel of water circulation, still under the influence of marine waters. This channel extends from the oceanic entrance of the bay to Paquetá. However, two opposite situations occur in this region: while the oceanic flow helps to remove nutrients and sediment, thereby enabling greater depth and also maintaining high levels of dissolved oxygen [33], which was identified in our study in 2017, its borderline position slows the speed of the tidal current that reaches the region [10]. Thus, this increase in oxygenation may be precisely related to an event that improved the water flow, promoting better oxygenation in the region and removal of sediments (for example sea currents and wind energy), boosting and improving the speed of the water flow of collection in 2017, which was not repeated in 2018, in relation to oxygenation. In addition, the dredging itself can be influenced in improving this factor, since all points showed an increase in oxygen concentration [11], even if subtle.

Regarding transparency, Urca and Flamengo exhibited more transparent waters than Paquetá and Engenho after dredging. As transparency is influenced by the amount of dissolved organic matter, the regions closest to the mouth of Guanabara Bay tend to be more transparent due to the influence of the tidal regime and the dilution of marine water. Chaves et al. [27] and Souza et al. [34] also reported the same transparency gradient, with Urca being more transparent than Paquetá.

The dredging process had little influence on pH levels, due to the natural composition of GB water [27,34] and according to Choppala et al. [35] who reported the same results during the dredging process.

Salinity and temperature also did not vary significantly during the study period between the sampling points, and the salinity values were in accordance with Fistarol et al. [10] for the region, ranging from 13 to 36 g L^−1^ [27,34]. No salinity gradient was observed, contrary to what was reported by Seixas et al. [13], and the temperature values were also discrepant in relation to the expectation of warmer internal waters compared to the external areas, which are influenced by the ocean [10].

The PCA analysis corroborated the results identified in the analysis of means. Particularly in relation to the port region, the PCA indicates low variations over the study period. Inverse correlations were observed between temperature and oxygen and depth, as deeper environments have lower concentrations of temperature and oxygen, as expected [17]. However, Chaves et al. [27] reported that temperature was negatively correlated with salinity, transparency, pH and oxygen.

The dredging process was expected to lead to several benefits, mainly in the port region of Rio de Janeiro, although adjacent regions may also reflect changes identified in the port area, due to the removal of sediment and organic matter. In addition, the process of macro-draining surface waters would lead to improvements in access to ocean water to the bay, which would lead to a better supply of oxygen, as previously mentioned, in addition to an increase in salinity and pH and a decrease in temperature [10,11,27,34]. However, in the study none of these factors varied significantly as expected when comparing the before and after dredging, only the increased transparency in Urca and Flamengo, as we justified earlier.

### 4.2. Factors Affecting Hg Concentrations

The THg concentrations in animals residing in the port region of Rio de Janeiro before and after the dredging process were not statistically significant. Swimming crabs were not captured during the dredging period, which is probably directly related to the dredging process, since these animals are benthic and remain in direct contact with the sediment. As dredging removes sediment, it is assumed that this activity removes part of the local fauna and/or disperses the animals to adjacent areas.

Significant differences in relation to THg concentrations during the three dredging periods were observed in the evaluation of animals present in the adjacent port points. The increase in concentration during dredging is related to the increase in the bioavailability of Hg, being more absorbed by animals due to the resuspension of sediments, caused mainly by its mechanical removal promoted by dredging, as well as by events that normally happen as currents or waves that exert a shear force in the sediment [9]. The resuspended Hg can be complexed with particulate material, such as organic matter and other substances and settles at the bottom, so at that moment minimal concentrations are observed and consequently the reduction in the bioavailability of the metal [9], which was observed in the post dredging period (2018). Unfortunately, no additional monitoring was carried out in the following months; therefore, it was not possible to verify whether there were changes in THg concentrations or whether it remained stable. Based on the three-year balance, our hypothesis that dredging would reduce the THg concentration in animals has not been confirmed. On the contrary, there was an increase in THg concentrations comparing before and after dredging. Possibly two factors are responsible for the increase in the concentration of mercury in animals: first, as mentioned earlier, the Guanabara bay is the target of numerous polluting sources of mercury (for example, industrial and domestic sewage disposal), in addition, the sedimentation rate is high [10], probably, even with the removal of part of the contaminated sediment, the constant discharge of the metal in this estuary maintains the level of contamination. The second issue is related to the bioavailability of the metal, due to the resuspension of the sediment during the process. Consequently, with greater availability, more metal was absorbed by the animals’ breathing and feeding, accumulating in the tissues. This effect of bioaccumulation, higher than the rate of excretion, means that the tendency is always to increase the levels of metal in living organisms, especially in places where the source of contamination is constant, such as BG. If, in fact, the dredging had an effect, in the post-dredging period we would observe at least concentrations equal to those of the previous year or due to the period of one year, they would show downward trends. What would be different, for example, if measurements were made in water. In this case, the difference between the three evaluated moments would probably be evident.

Some expected correlations can be made with abiotic factors. The depth was correlated with temperature and Hg according to the RDA. Environments of less depth tend to have a higher temperature and the heat promotes the acceleration of animal metabolism, thus the respiration rate will be higher and consequently the absorption via respiration of metals will increase [36], the opposite being true. However, the oxygen ratio seems to diverge from what was expected, despite the three factors (oxygen, depth and temperature) showing coherent relationships with each other, when confronted with Hg concentrations, they presented a different behavior than expected. Studies show higher Hg concentrations in more anoxic environments. This is because the lower concentration of dissolved oxygen also stimulates the higher rate of respiration and consequently the uptake of Hg from water. Also, according to Sadhu et al., [37] deep waters have higher decomposition rates and lower oxygen content, factors that contribute to the production of methylmercury, since sulfate-reducing bacteria have a predilection for anaerobic environments. Although we have not gauged this form of mercury, studies indicate that almost 100% of the Hg that is captured and accumulated in the animal is in this form, which has high bioavailability, in addition to being more absorbable and bioaccumulative [6,38,39,40].

In relation to the other factors not correlated in the RDA directly with Hg, but which also influence its presence, bioavailability, methylation and absorption, the low transparency (except in Urca and Flamengo) is related to the high content of dissolved organic matter, which it increases methylation, as it provides more carbon for sulfate-reducing bacteria [41]. During dredging, when we see the highest concentration of mercury in animals, all points have reduced transparency. This can happen both because of the high content of organic matter due to pollution of the bay, and mainly because of the re-suspension of the sediment, which promotes the reduction of transparency.

Lower salinity was observed mainly after dredging, a period when the concentration of mercury remained high compared to the period prior to dredging (2016). It is expected that environments with low salinity present high levels of Hg, this because there is less competition of sodium ions with the metal, favoring its absorption by the animal, unlike the environment with higher salinity, where due to this competition the absorption of metals is in disadvantage [36,42,43,44]. In addition, in relation to the methylation process, it is reduced in brackish water. This may occur due to the bonding of the sulfide present in salt water with inorganic Hg, making it less bioavailable for the methylation process [42]. The high salinity can also negatively interfere with the activity of sulfate-reducing bacteria due to the increased sensitivity of some species to salinity, leading to a lower methylation rate [45]. Regarding pH, a slightly alkaline pH was observed here, but acidic environments are more conducive to the absorption of metals, since, with a lower pH, Hg becomes more bioavailable [41,46].

Comparing the external and internal points, although we did not identify any statistical difference between collection points, it is possible to highlight that the internal region of the bay (Engenho and Paquetá) showed the greatest difference between before and after. In 2016 the animals showed low concentrations of mercury, while in 2018 they doubled that value. As previously mentioned, this region has peculiar characteristics, especially Paquetá as previously mentioned. Both areas are heavily impacted by local pollution [10], a fact evidenced by the low transparency in the regions, which may justify the high contamination.

### 4.3. Risk to Consumption

Swimming crabs are in close contact with the contaminated sediment, which could favor Hg bioaccumulation. However, they feed on other organisms belonging to the same trophic level, leading to low mercurial biomagnification [16]. These factors lead to variable Hg concentrations in these animals, and the influence of abiotic factors may be high. The Hg concentrations observed here are compatible with other studies that address the concentration of mercury in swimming crabs that inhabit estuaries, such as Guanabara Bay, with our lowest value found being 0.052 and the highest 0.155 Hg mg kg^−1^ w.w. Our previous study found concentrations between 0.103 and 0.100 Hg mg kg^−1^ w.w. in swimming crabs in 2015 and 2016 in the outer region (Urca) of the bay, a value similar to what we found in the present study (0.097 to 0.110 Hg mg kg^−1^ w.w.) [5]. Bordon et al. [26] concentrations between 0.16 and 0.32 Hg mg kg^−1^ d.w. (0.04 to 0.08 Hg mg kg^−1^ w.w.) in the study of swimming crabs in the Santos estuarine system, São Paulo, Brazil; Taylor and Calabrese [6] found in Narraganset Bay (Rhode Island/ Massachusetts, USA) 0.208 to 0.226 Hg mg kg^−1^ d.w. (0.052 to 0.0565 Hg mg kg^−1^ w.w.); Olivero-Verbel et al. [21] found in swimming crabs of Cartagena Bay values of 0.498 Hg mg kg^−1^ d.w. of mercury (0.124 Hg mg kg^−1^ w.w.). Reichmuth et al. [24] found in its study in Mullica River-Great Bay estuary the concentration of approximately 0.15 Hg mg kg^−1^ d.w. (0.037 Hg mg kg^−1^ w.w.) and in Hackensack Meadowlands-Hudson-Raritan estuary the value of approximately 0.45 Hg mg kg^−1^ d.w. (0.112 Hg mg kg^−1^ w.w.). Based on these studies, we see that our results are within the range of values found elsewhere, but also in estuarine environments and with animals of the same species. These results are probably related to the habitat of these animals, which in constant contact with the sediment, are more prone to mercury contamination and bioaccumulation.

Although all Hg values in the assessed swimming crabs were below the stipulated limit of 1.0 mg kg^−1^ THg in seafood determined by the European Union and 0.5 mg kg^−1^ [47] in crustaceans by the Brazilian regulatory agency ANVISA [48]. However, Hg concentrations lower than stipulated limits do not necessarily mean no risks [49], as limits exist mainly to guide decision-making, generating alerts so early measures may be adopted to prevent negative human health effects. However, as Hg bioaccumulates even when present in small doses, its effects will be observed in the long term, especially if the consumption frequency of a contaminated food item is high [49]. Although the goal of this study was to evaluate only THg concentrations, it is important to note that many studies point out that most Hg content found in aquatic animals is in its organic form, MeHg, the most toxic for of this element [6,38,39,40].

Thus, the maximum limits stipulated for human Hg intake per week should be taken into account. The provisional tolerable weekly intake (PTWI) for THg is of 4 μg kg^−1^ b.w./week according to the JECFA/WHO [50], while the (US) National Research Council (NRC) has established an intake limit of 0.7 μg kg^−1^ b.w./week [51]. Weekly Hg levels were, thus, calculated based on the annual average consumption of seafood reported for the metropolitan region of Rio de Janeiro of 18.5 kg [18], or a weekly consumption of 0.356 kg. The results reported in Table 2 indicate that all swimming crab THg consumption levels were within the stated guidelines for all sampling locations and periods. However, it is clear that during dredging, THg values were increased at the internal and external areas (no animals were found at the port in 2017, during the dredging periods), although no statistical analyses could be carried out. In addition, Hg intake concentrations per week during the post-dredging period were slightly increased compared to the pre-dredging period, seemingly corroborating Hg resuspension and increased bioavailability, probably due to methylation by sulfate-reducing anaerobic bacteria, forming MeHg [6,7,8]. Even though swimming crabs presented lower THg concentrations than the stated guidelines, as they are significantly and constantly consumed around Guanabara Bay, public health issues may arise in the long term, and continuous THg monitoring of these organisms is indicated to carry out human and biota risk assessments. These results may also serve as basis for environmental risk assessment in other areas undergoing current or future dredging activities, both in Brazil and worldwide.

## 5. Conclusions

Our study identified that dredging did not have the expected effect either in the Porto region, which despite not having been evaluated during the process, showed similar initial and final mercury values, as in the adjacent areas. In these adjacent areas, where we were able to investigate the concentration of mercury during the dredging period, we observed an increase in Hg values. This indicates that the process may have had repercussions in all the evaluated points of the bay and added to events not related to dredging, but that may also promote the resuspension of the contaminated sediment or that may favor water contamination or even greater metal absorption. Evaluating the panorama of the contamination of the three studied periods, the THg values found in the study were increased during the dredging apparently due to the resuspension of Hg present in the contaminated sediment and increasing the bioavailability of the metal, and reduced in the following year, however to values still higher than the initial ones. Additional assessments on these organisms would be needed to see if concentrations continue to decline or remain stable, establishing long-term dredging effects. Possibly the justification is the constant contamination of the bay, which can overcome the effects of processes such as dredging, in addition to the effect of bioaccumulation of mercury in animal tissues. Regarding the abiotic factors investigated in Guanabara Bay, the study pointed out that metal concentrations in animals were directly positively related to temperature and oxygen content and negatively to depth. Highlighting the temperature, which favors a higher respiratory rate and mercury uptake via respiration. In addition, transparency is another factor that can be highlighted despite not having a direct correlation with Hg. We have identified low transparency in most of the bay, probably due to the mobilization of sediments by dredging, but also being an indication of the large amount of organic matter dispersed in the water as a result of the contamination of this estuary.

Although THg levels are below the maximum permitted limits for mercury in crustaceans and the weekly intake calculations also point to the absence of health risk, it is important to note that these animals are consumed continuously and significantly in Guanabara Bay, and that the metal bioaccumulates in tissues throughout life, which can lead to long-term public health problems.

## Figures and Tables

**Table 1:** Abiotic variables of each sampling point, before, during and after Rio de Janeiro sediment dredging.

Variable	Area	Period		
		Before	During	After
Depth (m)	Urca	9.00 ± 1.41	7.50 ± 2.47	7.50 ± 2.47
	Flamengo	11.00 ± 4.24	10.60 ± 0.84	10.60 ± 0.84
	Seaport	7.00 ± 0.00	6.50 ± 0.00	6.50 ± 0.00
	Engenho	10.75 ± 5.30	8.25 ± 0.35	8.25 ± 0.35
	Paquetá	15.00 ± 2.82	16.50 ± 6.36	16.50 ± 6.36
Transparency (m)	Urca	1.25 ± 0.07	0.85 ± 0.07	4.45 ± 2.19
	Flamengo	0.85 ± 0.21	1.55 ± 0.07	4.25 ± 0.35
	Seaport	0.90 ± 0.14	1.05 ± 0.32	1.05 ± 0.07
	Engenho	0.85 ± 0.07	0.30 ± 0.14	0.80 ± 0.00
	Paquetá	1.00 ± 0.00	0.58 ± 0.10	0.80 ± 0.00
pH	Urca	8.49 ± 0.03	8.30 ± 0.08	8.51 ± 0.21
	Flamengo	8.75 ± 0.01	8.16 ± 0.12	8.48 ± 0.13
	Seaport	8.58 ± 0.06	8.01 ± 0.09	8.45 ± 0.15
	Engenho	8.49 ± 0.03	7.95 ± 0.50	8.36 ± 0.15
	Paquetá	8.05 ± 0.27	7.98 ± 0.06	8.67 ± 0.13
Oxygen (mg L^−1^)	Urca	5.26 ± 0.50	9.84 ± 0.29	5.99 ± 0.50
	Flamengo	5.74 ± 0.25	8.41 ± 0.17	6.80 ± 1.93
	Seaport	4.24 ± 0.03	6.07 ± 0.24	4.36 ± 0.73
	Engenho	3.20 ± 0.97	4.64 ± 0.22	4.05 ± 0.08
	Paquetá	3.18 ± 0.12	8.54 ± 3.72	3.07 ± 1.05
Salinity (g L^−1^)	Urca	32.67 ± 0.59	31.72 ± 0.16	28.97 ± 0.36
	Flamengo	32.80 ± 0.10	32.54 ± 0.35	29.79 ± 0.10
	Seaport	31.17 ± 0.55	31.86 ± 0.25	28.38 ± 0.39
	Engenho	31.72 ± 1.39	31.81 ± 0.29	29.14 ± 0.33
	Paquetá	32.66 ± 0.02	32.10 ± 0.41	28.60 ± 0.84
Temperature (°C)	Urca	23.76 ± 0.55	25.32 ± 0.007	24.96 ± 0.31
	Flamengo	23.96 ± 0.23	25.18 ± 0.14	24.77 ± 0.06
	Seaport	24.88 ± 0.36	25.57 ± 0.10	24.25 ± 0.46
	Engenho	23.91 ± 1.01	24.81 ± 0.10	23.50 ± 0.24
	Paquetá	23.00 ± 0.06	24.87 ± 0.13	23.93 ± 1.11

**Table 2:** Mean values and standard deviation of THg (mg kg^−1^) w.w. in swimming crabs, collected in the internal, Porto and external points of Guanabara bay, before, during and after dredging.

Collect Points	THg before Dredging	THg during Dredging	THg after Dredging
Internal Points (Engenho and Paquetá)	0.052 ± 0.020	0.111 ± 0.052	0.108 ± 0.008
Seaport	0.098 ± 0.114	-	0.111 ± 0.069
External Points (Urca and Flamengo)	0.097 ± 0.004	0.155 ± 0.06	0.110 ± 0.025

**Table 3:** Human Hg intake per week in the internal, port and external sampling locations assessed herein. Data are expressed as mg kg bw/week.

Location	2016	2017	2018
Internal areas	0.034	0.055	0.039
Port	0.035	-	0.040
External areas	0.018	0.039	0.038
